# Low prevalence of SARS‐CoV‐2 specific antibodies among endoscopists and their assistants in a university hospital in Tochigi prefecture–A single‐center study

**DOI:** 10.1002/deo2.79

**Published:** 2021-12-03

**Authors:** Kouichi Miura, Hiroshi Maeda, Naoshi Arai, Mariko Sekiya, Akihiro Miyano, Masako Watanabe, Keijiro Sunada, Koichi Hagiwara, Yuji Morisawa, Toshiyuki Yamada, Naohiro Sata, Alan Kawarai Lefor, Tatsuhiko Kodama, Ryozo Nagai, Hironori Yamamoto

**Affiliations:** ^1^ Department of Medicine, Division of Gastroenterology Jichi Medical University Tochigi Japan; ^2^ Department of Medicine, Division of Pulmonary Medicine Jichi Medical University Tochigi Japan; ^3^ Department of Infection and Immunity, Division of Infection disease Jichi Medical University Tochigi Japan; ^4^ Department of Clinical Laboratory Medicine Jichi Medical University Tochigi Japan; ^5^ Department of Surgery, Division of Gastrointestinal, General and Transplant Surgery Jichi Medical University Tochigi Japan; ^6^ Research Center for Advanced Science and Technology the University of Tokyo Tokyo Japan; ^7^ Jichi Medical University Tochigi Japan

**Keywords:** antibody, COVID‐19, endoscopy, healthcare worker, seroprevalence

## Abstract

**Objectives:**

Gastrointestinal endoscopic procedures have a risk to transmit severe acute respiratory syndrome coronavirus 2 (SARS‐CoV‐2) through aerosols. Little information is available on the seroprevalence of SARS‐CoV‐2 antibodies among healthcare workers (HCWs) in endoscopy units. Thus, the seroprevalence was examined in HCWs who do and do not participate in gastrointestinal endoscopy.

**Methods:**

A total of 382 HCWs at Jichi Medical University Hospital were enrolled in this study through March 2021. Among 382 HCWs, 63 are in the endoscopy unit. Serum antibody levels against SARS‐CoV‐2 were determined by immunochromatography, chemiluminescent immunoassay (CLIA), electric CLIA (ECLIA), and chemiluminescence microparticle immunoassay (CMIA). HCWs in the endoscopy unit underwent testing up to three times. We defined antibody‐positive as when at least one test was positive.

**Results:**

The seroprevalence of SARS‐CoV‐2 antibodies in 63 HCWs in the endoscopy unit was 0%–1.9%, 0%–1.7%, and 0%–1.7% during the first (Ap–May 2020), second (Jun–Nov 2020), and third intervals (Dec 2020–Mar 2021), respectively. This seroprevalence was comparable to that of other HCWs not involved with gastrointestinal endoscopy. Two HCWs in the endoscopy unit were positive for antibodies: one was ECLIA‐positive and the another was CMIA‐positive. The ECLIA‐positive HCW was PCR negative and converted to negative for the second and third tests. Another HCW was CMIA‐positive at all three evaluations and the titers were unchanged. No HCWs in the endoscopy unit contracted a SARS‐CoV‐2 infection.

**Conclusions:**

The seroprevalence of SARS‐CoV‐2 antibodies was low among HCWs in the endoscopy unit through March 2021 (UMIN000039997).

## INTRODUCTION

The spread of severe acute respiratory syndrome coronavirus 2 (SARS‐CoV‐2) continues to burden healthcare systems worldwide. It is estimated that over 200 million people contracted coronavirus disease‐2019 (COVID‐19) and four million died through the beginning of August 2021. In Japan, the cumulative numbers of patients with COVID‐19 were reportedly greater than one million at the beginning of August 2021. The stress on the entire healthcare system continues to increase because of the tremendous increase in the number of patients with COVID‐19.

One of the most serious problems caused by this infection is the potential collapse of healthcare systems due to the spread of infection to healthcare workers (HCWs).^1^ Once a cluster of COVID‐19 occurs among HCWs in a medical institution, patient care becomes much more difficult. As a result, medical care has contracted due to the decreased ability to interact with patients. Although medical institutions make efforts to prevent nosocomial infections, they are difficult to be eliminated. One reason for this is the presence of asymptomatic individuals who can transmit SARS‐CoV‐2. Polymerase chain reaction (PCR) screening is not always available for asymptomatic individuals. In addition, patients visit medical institutions without PCR screening. Thus, HCWs may become transmitters of SARS‐CoV‐2 within medical institutions without being aware.

Among HCWs, the risk of SARS‐CoV‐2 infection in those who directly care for patients was reportedly higher compared to HCWs who do not directly care for patients.^2^ HCWs in an endoscopy unit have a high risk of SARS‐CoV‐2 infection because the routine gastrointestinal endoscopic practice can generate aerosols which carry SARS‐CoV‐2. Indeed, SARS‐CoV‐2 infection of HCWs who work in endoscopy units was found to be 4.3% (42/968) in Northern Italy, an endemic area,^3^ and 14% (10/73) in Egypt, where the cumulative number of patients with COVID‐19 was equivalent to that of Japan.^4^ Although the seroprevalence of SARS‐CoV‐2 antibodies among HCWs was reported to be 0.07%–6.25% in Japan,^5^, ^6^, ^7^, ^8^, ^9^, ^10^ there are few data on the seroprevalence of SARS‐CoV‐2 antibodies among HCWs in endoscopy units. This study was undertaken to investigate the seroprevalence of SARS‐CoV‐2 antibodies among HCWs who practice and support gastrointestinal endoscopy. In addition, the seroprevalence in other HCWs who do not engage in gastrointestinal endoscopy practice was evaluated in our hospital, located in Tochigi prefecture, 90 km north of Tokyo. The density of people infected with SARS‐Cov‐2 in Tochigi prefecture was 2383/million at the end of March 2021.

## MATERIALS AND METHODS

### Participants

A total of 382 HCWs were enrolled in this study from April 2020 through March 2021. Among 382 HCWs, there are 63 HCWs, including 48 physicians and 15 nurses, who practice and/or support gastrointestinal endoscopy at least once per week. In the present study, we defined these 63 HCWs as HCW involved in gastrointestinal endoscopy. Doctors with less than 2‐year‐experience were excluded. Among 48 physicians, 39 (81%) performed endoscopic examinations outside of our hospital. Seven physicians (15%) infrequently had work related to SARS‐Cov‐2 infection, including nasopharyngeal sampling for PCR screening before surgery. HCWs working in the endoscopy unit underwent SARS‐Cov‐2 antibody testing at the most three times during the study period. The present study also included HCWs at high risk of SARS‐Cov‐2 infection: a total of 24 HCWs who potentially see patients with COVID‐19 (eight HCWs from respiratory division and 16 HCWs from otolaryngology division) and a total of 23 HCWs who see patients with severe COVID‐19 in the intensive care unit.

This study was approved by the Institutional Review Board (A20‐065) and registered at the University Hospital Medical Information Network Clinical Trials Registry as UMIN000039997. The study was performed according to the ethical guidelines of the Declaration of Helsinki.

### Detection of SARS‐Cov‐2 antibodies

SARS‐Cov‐2 antibody tests were performed using four methods, including immunochromatography (IC), chemiluminescent immunoassay (CLIA), electric CLIA (ECLIA), and chemiluminescence microparticle immunoassay (CMIA) methods. To perform the IC method, we used three different kits due to shortages, including the SARS‐Cov‐2 antibody detection kit (Cat No. RF‐NC001, RF‐NC002; Kurabo, Osaka, Japan), 2019‐nCoV IgG/IgM Rapid Test Cassette (Cat No. INCP‐402; MedNet GmbH, Muenster, Germany), and the 2019‐nCoV Ab Test (Innovita Biological Technology Hebei, China), according to the manufacturers’ instructions. These kits were used for rapid detection. In addition, we quantified antibody titers using CLIA (iFLASH; YHLO Biotech, Shenzhen, China), ECLIA methods (Elecsys; Roche Diagnostics, Basel, Switzerland), and CMIA (SARS‐Cov‐2 IgG II Quant; Abbott, Chicago, IL, USA). Rapid tests were done immediately after blood collection. The remaining serum was stored at –80°C until the CLIA, ECLIA, and CMIA assays were performed. Cut‐off values to determine positive tests were as follows: ≥10 for CLIA, ≥1.0 for ECLIA, and ≥1.0 for CMIA. A polymerase PCR test was performed for individuals suspected to have a SARS‐Cov‐2 infection. We defined antibody‐positive as being when at least one test was positive in the present study.

### Endoscopy unit procedures and precaution for SARS‐CoV‐2

**FIGURE 1:**
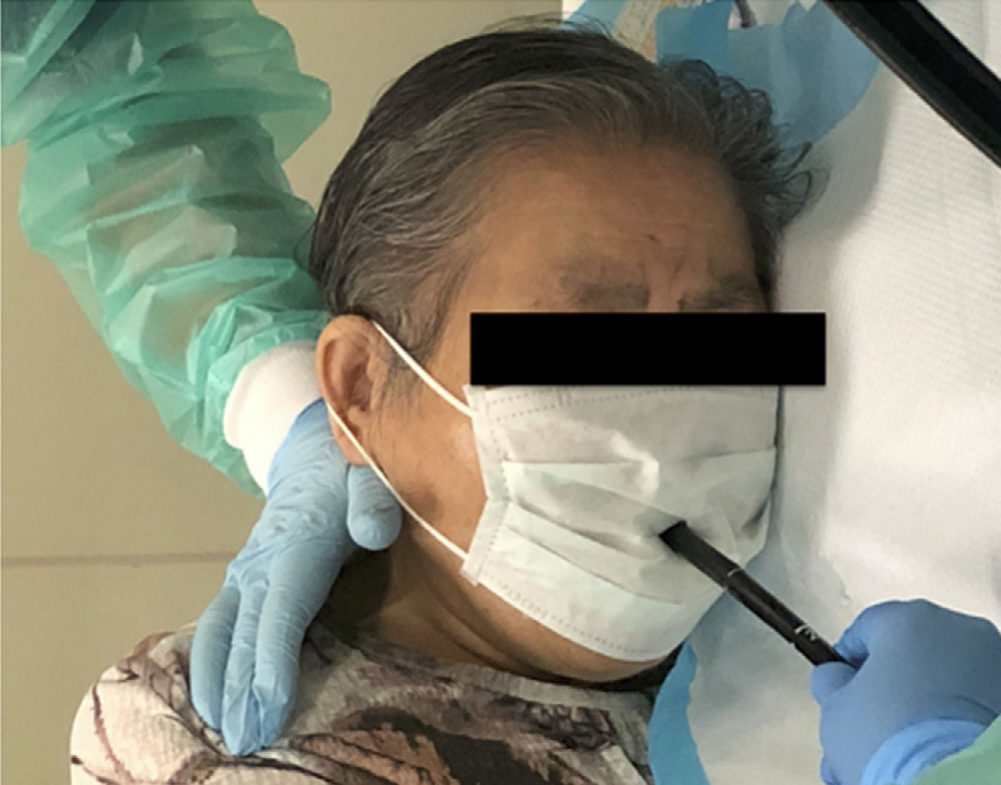
A patient undergoing esophagogastroduodenoscopy while wearing a mask

This endoscopy unit performs esophagogastroduodenoscopy, colonoscopy, double‐balloon enteroscopy, and endoscopy for hepato‐biliary‐pancreatic diseases. The volume of endoscopy practice was obtained from the records of the endoscopic unit. More than 95% of endoscopic procedures were performed by 48 physicians who participated in the present study. Before undergoing endoscopy, patients reported their health condition using a questionnaire (Table S1). If patients had a temperature >37.5°C and reported “yes” for potential infection with SARS‐Cov‐2, the endoscopy was rescheduled except for emergency procedures. PCR screening for SARS‐Cov‐2 for patients scheduled for invasive gastrointestinal endoscopic procedures (endoscopic submucosal dissection, endoscopic variceal ligation, etc.) was started on June 1, 2020. Disposable masks with a hole are reported to reduce aerosols during endoscopic examinations.^11^, ^12^ Patients wore the mask during endoscopic examinations starting in January 2021 (Figure 1). Before the COVID‐19 pandemic, endoscopists and endoscopy nurses wore scrub clothing, masks, and disposable aprons, exchanging the apron for every patient. After the outbreak of COVID‐19 in Japan, endoscopists and nurses perform endoscopy according to the guideline from the Japan Gastroenterological Endoscopy Society. However, a sufficient supply of personal protective equipment was not guaranteed, and gowns were exchanged every 4 h. These modified precautions were started in the endoscopy unit on April 8, 2020. Personal protective equipment for SARS‐CoV‐2 in this hospital is shown in Table S2.

### Number of people infected with SARS‐Cov‐2

The number of people infected with SARS‐Cov‐2 at Jichi Medical University Hospital, Tochigi prefecture, and other areas were obtained from websites maintained by Jichi Medical University (www.jichi.ac.jp), the Tochigi prefectural (www.pref.tochigi.lg.jp/e04/welfare/hoken‐eisei/kansen/hp/coronakensahasseijyoukyou.html; accessed July 18, 2021), and the Japanese Government (www.mhlw.go.jp/stf/seisakunitsuite/newpage_00016.html; accessed September 22, 2021) and World Health Organization (https://covid19.who.int; accessed September 22, 2021), respectively. The density was calculated as follows: cumulative people infected with SARS‐Cov‐2 by population in April 2020.

### Statistical analysis

Differences between the two groups were evaluated for statistical significance using the chi‐squared test (GraphPad Prism 8.4.2; GraphPad Software Inc, San Diego, CA). A *p*‐value less than 0.05 was defined as statistically significant.

## RESULTS

### Number of people infected with SARS‐Cov‐2 in each country and area

**FIGURE 2:**
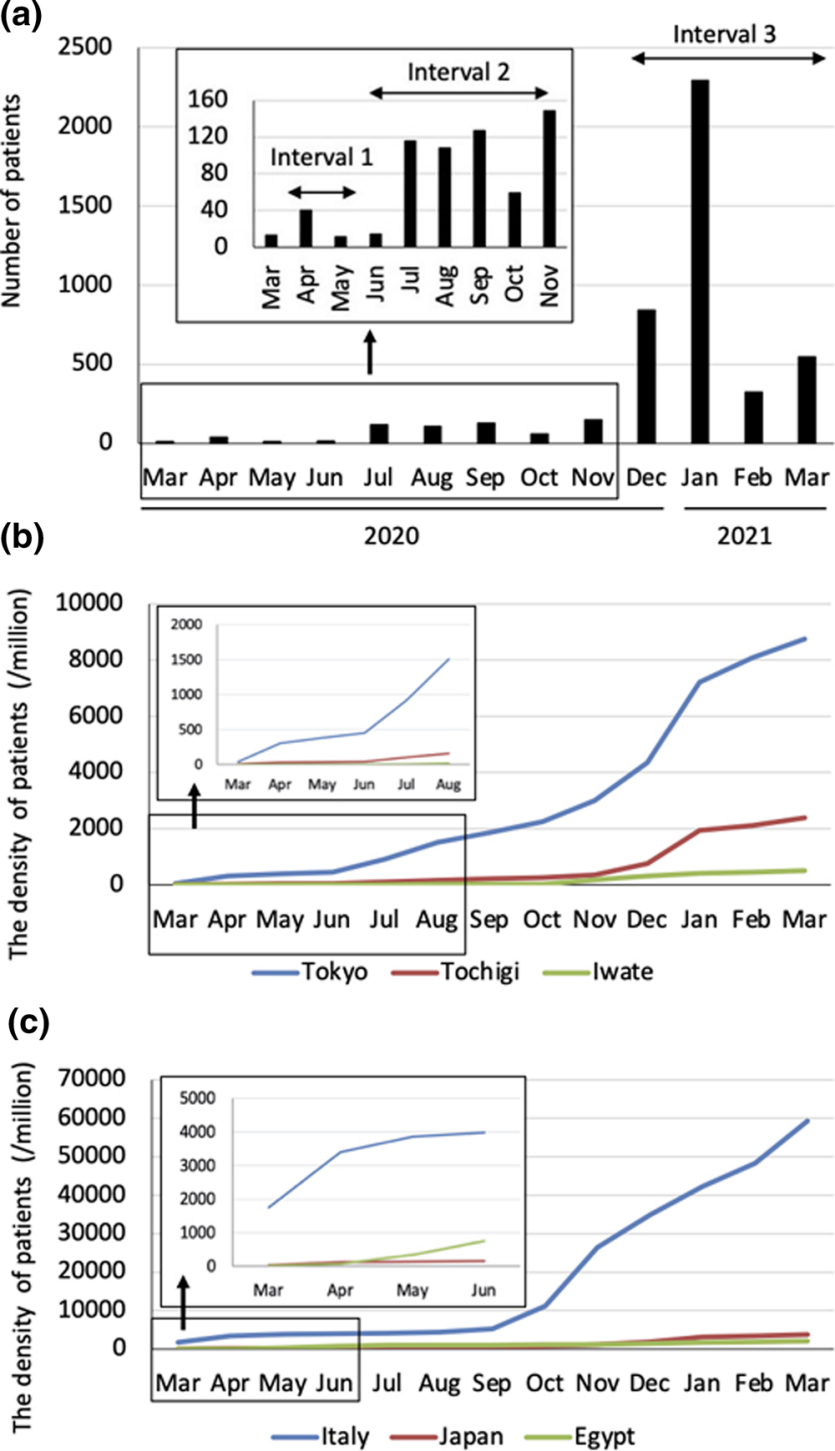
(a) The number of people infected with severe acute respiratory syndrome coronavirus 2 (SARS‐CoV‐2) in Tochigi prefecture, with an expanded view of the time period March–November 2020 (Inserted). We divided the study period into three intervals according to the wave of coronavirus disease‐2019 (COVID‐19). (b) The density of people infected with SARS‐CoV‐2 in Tokyo, Tochigi, and Iwate prefectures, with an expanded view of the time period March–August 2020 (Inserted). Study periods were May 29–31 and June 6–August 21 performed in Iwate^5^ and Tokyo,^6^ respectively. (c) The density of people infected with SARS‐CoV‐2 in Italy, Japan, and Egypt, with an expanded view of the time period March–June 2020 (Inserted). Study periods were Jan 27–March 13 and June 1–14 performed in Italy^3^ and Egypt,^4^ respectively

Figure 2a shows the number of people infected with SARS‐Cov‐2 in Tochigi prefecture where the hospital is located. The number of people newly diagnosed with SARS‐Cov‐2 infection increased in April 2020 and then decreased after “the first declaration of a state of emergency” in May 2020 by the Japanese Government. The number of people infected with SARS‐Cov‐2 increased again from July to September 2020 and then increased again from November 2020 to January 2021 (Inserted Figure). The maximum number of people infected with SARS‐Cov‐2 in Tochigi prefecture was 47.2/million on January 10, 2021, and the maximum positivity rate of the PCR test was 10.1% on January 12, 2021. People infected with SARS‐Cov‐2 were noted to be diffusely distributed in Tochigi prefecture, including the southern region of the prefecture where Jichi Medical University Hospital is located.

Then, we investigated the number of people infected with SARS‐Cov‐2 in Japan and other countries, where seroprevalence data and infected cases in HCWs were available. Figure 2b includes the density of people infected with SARS‐Cov‐2 in Tokyo and Iwate prefectures, where the seroprevalence of HCWs by Roche assay was 0.34%^6^ and 0.07%,^5^ respectively. Figure 2c includes the density of people infected with SARS‐Cov‐2 in Italy and Egypt. The density of Japan was far lower than that of Italy but equivalent to Egypt.

From April 1, 2020 through March 2021, 66 patients with COVID‐19 were hospitalized at Jichi Medical University Hospital. During a screening for SARS‐Cov‐2 infection, three patients (0.049%) were PCR positive among 6127 patients who had a PCR test before surgery or hospitalization.

Information regarding the number of HCWs who had SARS‐Cov‐2 infection at Jichi Medical University Hospital was not released. However, one HCW in the Emergency Care Unit was infected with SARS‐Cov‐2 at the beginning of September 2020 (A press release from the hospital but now deleted). This person worked in the hospital before being diagnosed with SARS‐Cov‐2 infection. There were 112 HCWs identified around that worker, and all were PCR negative. A cluster of COVID‐19 did not develop during the study period.

### Seroprevalence of SARS‐CoV‐2 antibodies among HCWs

During the study period, two HCWs in the endoscopy unit had a positive result for any of the antibody tests used. The seroprevalence among HCWs in the endoscopy unit was 0%–1.9% during the first interval, 0%–1.7% during the second interval, and 0%–1.7% during the third interval (Table 1). One physician was positive by ECLIA during the first interval but negative by the other three methods, and then converted to a negative result during the second and third intervals. One nurse was positive during all three intervals only by the CMIA test but not using other methods. Although two physicians performed esophagogastroduodenoscopy on two patients with COVID‐19 during the study period, they had negative antibody tests after the procedures.

**TABLE 1:** Seroprevalence of severe acute respiratory syndrome coronavirus 2 (SARS‐CoV‐2) antibodies in healthcare workers in endoscopy and non‐endoscopy units

	**First interval (Apr–May 2020)**					**Second interval (Jun–Nov 2020)**					**Third interval (Dec 2020**–**Mar 2021)**				
**Endoscopy units**	***n***	**IC**	**CLIA**	**ECLIA**	**CMIA**	***n***	**IC**	**CLIA**	**ECLIA**	**CMIA**	***n***	**IC**	**CLIA**	**ECLIA**	**CMIA**
Total	54	0	0	1 (1.9%)	1 (1.9%)	58	0	0	0	1 (1.7%)	57	0	0	0	1 (1.7%)
Physicians	39	0	0	1 (2.6%)	0	43	0	0	0	0	44	0	0	0	0
Nurses	15	0	0	0	1^a^ (6.7%)	15	0	0	0	1^a^ (6.7%)	13	0	0	0	1^a^ (7.7%)

	**First interval (Apr–May 2020)**					**Second interval (Jun–Nov 2020)**					**Third interval (Dec 2020–Mar 2021)**				
**Non‐endoscopy units**	***n***	**IC**	**CLIA**	**ECLIA**	**CMIA**	***n***	**IC**	**CLIA**	**ECLIA**	**CMIA**	***n***	**IC**	**CLIA**	**ECLIA**	**CMIA**
Total	0	NE	NE	NE	NE	120	1^b^ (0.8%)	1^b^ (0.8%)	0	1^b^ (0.8%)	26	0	0	0	0
Physicians	0	NE	NE	NE	NE	39	0	0	0	0	7	0	0	0	0
Nurses	0	NE	NE	NE	NE	81	1^b^ (1.3%)	1^b^ (1.3%)	0	1^b^ (1.3%)	19	0	0	0	0

	**First interval (Apr–May 2020)**					**Second interval (Jun–Nov 2020)**					**Third interval (Dec 2020–Mar 2021)**				
Other HCWs	0	NE	NE	NE	NE	170	0	3^c^ (1.8%)	0	1^c^ (0.6%)	3	0	0	0	0

Abbreviations: CLIA, chemiluminescent immunoassay; CMIA, chemiluminescence microparticle immunoassay; ECLIA, electric CLIA; HCW, healthcare worker; IC, immunochromatography; NE, not examined.

^a^ One nurse was positive for antibody at all three tests by CMIA.

^b^ One nurse shown positive by three methods.

^c^ One HCW shown positive by two methods.

We also examined the seroprevalence of SARS‐CoV‐2 antibody in other HCWs who are not involved with gastrointestinal endoscopy (Table 1). The seroprevalence in these physicians and nurses was 0%–0.8%. The seroprevalence of other HCWs, including medical coworkers and clerical staff, was 0%–1.8% during the second interval. There were no significant differences in the seroprevalence in the endoscopy unit compared with non‐endoscopy units for the IC, CLIA, and CMIA tests. Among 120 HCWs in the second interval, 24 belong to the respiratory division (eight physicians) or otolaryngology division (eight physicians, four nurses, and four other HCWs) who potentially see patients infected with SARS‐CoV‐2. Although one HCW (4.2%, 1/24) was positive for the CLIA method, no statistical difference was noted when compared to the seroprevalence in the endoscopy unit. In the third interval, a total of 23 HCWs (four physicians and 19 nurses) who treated patients with severe COVID‐19 in the intensive care unit were included and their seroprevalence was 0% for any test used.

### Positive SARS‐CoV‐2 antibody tests in HCWs

A total of six HCWs were positive for SARS‐CoV‐2 antibody using any of the methods employed. The seroprevalence in each method was 0.26% (1/382), 0.26% (1/382), 0.76% (3/382), and 1.05% (4/382) in ECLIA, IC, CMIA, and CLIA method, respectively. The titer of ECLIA‐positive (Apr 2020) was just above the cut‐off value and then gradually decreased. In addition, the PCR test using a nasopharyngeal swab obtained on the same day was negative. One CMIA‐positive subject (Apr 2020) also tested positive during the second interval (Jul 2020) and the third interval (Mar 2021), during which the titer levels remained similar to the first one. Among six HCWs, two were simultaneously positive using multiple testing methods and the remaining four were positive by a single method (Table 2). No subjects were both positive using the ECLIA and CMIA methods, which was defined as “antibody positive” in a survey conducted by the Japanese Government. The CLIA‐ or CMIA‐positive subjects did not show a high titer as determined by the ECLIA method. Similarly, ECLIA‐positive subjects had a low titer by the CLIA or CMIA methods. In these six subjects, no symptoms were reported and their households had no individuals infected with SARS‐Cov‐2.

**TABLE 2:** Healthcare workers who tested positive for severe acute respiratory syndrome coronavirus 2 (SARS‐CoV‐2) antibodies

		**First interval (Apr–May 2020)**					**Second interval (June–Nov 2020)**					**Third interval (Dec 2020–March 2021)**			
**No**.	**HCW**	**IC**	**CLIA**	**ECLIA**	**CMIA**	**PCR**	**IC**	**CLIA**	**ECLIA**	**CMIA**	**PCR**	**IC**	**CLIA**	**ECLIA**	**CMIA**
1	Endoscopy unit	N	N (0.42)	P (1.43)	N (0.01)	N	N	N (0.48)	N (0.761)	N (0.01)	NE	N	N (0.37)	N (0.306)	N (0.01)
2	Endoscopy unit	N	N (0.36)	N (0.0846)	P (3.48)	NE	N	N (0.34)	N (0.0819)	P (3.15)	NE	N	N (0.35)	N (0.0818)	P (3.14)
3	Non‐endoscopy unit						P	P (34.41)	N (0.238)	P (1.88)	N				
4	Other HCW						N	P (21.25)	N (0.177)	NE	NE				
5	Other HCW						N	P (13.36)	N (0.082)	NE	NE				
6	Other HCW						N	P (23.30)	N (0.137)	NE	NE				

Abbreviations: CLIA, chemiluminescent immunoassay; CMIA, chemiluminescence microparticle immunoassay; ECLIA, electric CLIA; HCW, healthcare worker; IC, immunochromatography; N, negative; NE, not examined; P, positive; PCR, polymerase chain reaction.

Other HCWs include medical co‐workers and clerical staff in the non‐endoscopy unit. (titer); Positive was defined ≥10 for CLIA, ≥1.0 for ECLIA, and ≥1.0 for CMIA.

### Endoscopic procedures during the endemic of COVID‐19

**FIGURE 3:**
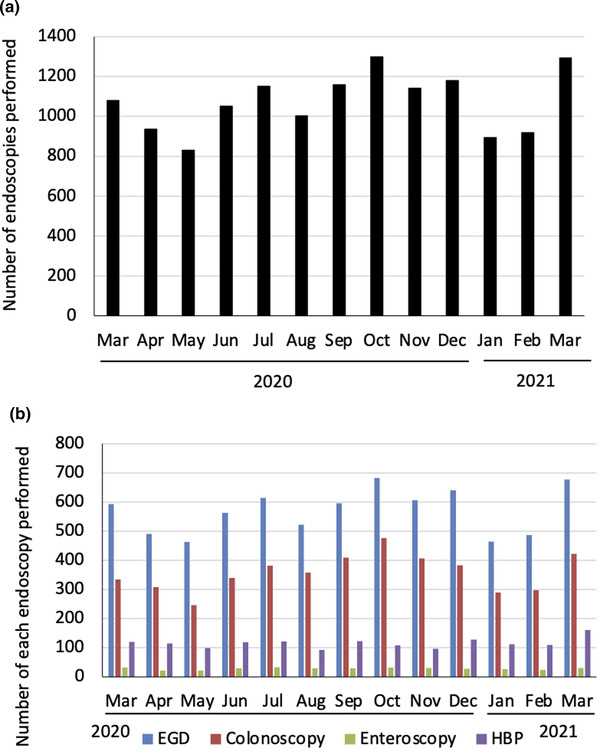
(a) The total number of endoscopic procedures performed in the endoscopy unit during the study period. (b) The number of each endoscopic procedure. EGD, esophagogastroduodenoscopy; HBP, endoscopy for hepato‐biliary‐pancreatic diseases

During the study, we performed endoscopy procedures according to statements released by medical associations, including the Japan Gastroenterological Endoscopy Society (www.jges.net/medical). Although the number of endoscopic examinations decreased by 20% (888 examinations) in May 2020 and by 16% (896 examinations) in January 2021 compared with those in 2019 and 2020, the clinical endoscopic practice was maintained at approximately 1000 procedures/month (Figure 3a). Among endoscopic procedures performed during the study period, about 50% were esophagogastroduodenoscopy and 10% were for hepato‐biliary‐pancreatic diseases (Figure 3b). Endoscopy was deferred in 14 patients who complained of respiratory symptoms or had other risk factors for contracting SARS‐CoV‐2 infections during the study period.

## DISCUSSION

Our study shows that the seroprevalence of SARS‐CoV‐2 antibodies in HCWs who work in an endoscopy unit is 0%–1.9%, which is unchanged regardless of the wave of COVID‐19 from April 2020 through March 2021. In addition, the seroprevalence is comparable to other HCWs not engaged in endoscopy practice. These findings suggest that HCWs in an endoscopy unit may not be at high risk for SARS‐CoV‐2 infection when using appropriate protection, at least in areas where COVID‐19 does not have a high prevalence.

This study corroborates a report that no HCWs contracted SARS‐CoV‐2 infection through endoscopic practice released by the Japan Gastroenterological Endoscopy Society (www.jges.net/medical/covid‐19‐qs). The survey, performed from July 2020 through August 2020, included 552 institutions, with over 50% of the institutions having patients with COVID‐19. Although the survey was questionnaire‐based, the present study partially confirmed the findings by serological tests. In addition, the serological tests were performed three times, corresponding to each of the waves of COVID‐19. In January 2021, the number of people infected with SARS‐CoV‐2 dramatically increased in Tochigi prefecture. The seroprevalence among HCWs was unchanged although we continued endoscopy practice. Although data for the seroprevalence of SARS‐CoV‐2 antibodies among HCWs in endoscopy units in Japan is not available, 4.3%–13.5% of HCWs in endoscopy units contracted SARS‐CoV‐2 infection in Italy^3^ and Egypt^4^ at the beginning of the pandemic. Thus, we should keep in mind that endoscopic practices carry the risk of transmission of SARS‐CoV‐2 without appropriate protection.

The present study showed that seroprevalence did not differ between the endoscopy unit and other areas of the hospital. Currently, the seroprevalence of SARS‐CoV‐2 antibodies was reported to be 0.07%–6.25% among HCWs in Japan.^5^, ^6^, ^7^, ^8^, ^9^, ^10^ These studies also showed that the seroprevalence in HCWs did not differ between high‐ and low‐risk groups. Since seroprevalence largely depends on the assays used, we performed four assays to facilitate comparison with other studies. The seroprevalence examined by Roche and Abbott assays in the present study was 0.26% and 0.76%, respectively. The seroprevalence of HCWs in Japan was reported to be 0.07%–0.34% and 0.36%–0.43% in Roche and Abbott assays, respectively.^5^, ^6^, ^7^ Considering these results together, SARS‐CoV‐2 infection did not spread among HCWs in our hospital. In contrast to Japan, many HCWs at high risk contracted SARS‐CoV‐2 during an early phase of endemic (before April 2020) in foreign countries, including Italy.^3^, ^13^ In Japan, most seroprevalence studies of HCWs were started from the end of May 2020 when medical institutions have already prepared for SARS‐CoV‐2. These data suggest that the timing of studies partially contributes to the difference in seroprevalence in HCWs.

The low seroprevalence among HCWs in our endoscopy unit may be explained by the following: 1) few patients with COVID‐19 underwent endoscopy, 2) triage (questionnaires and temperature monitoring) and screening for SARS‐CoV‐2 (PCR testing) effectively isolated patients with COVID‐19, 3) the use of standard precautions started before the COVID‐19 epidemic, and 4) patients wore masks while undergoing endoscopy, which may reduce the spread of aerosols.^11^, ^12^ Although the number and density of people infected with SARS‐CoV‐2 in Tochigi prefecture are small compared with Tokyo, three patients (0.049%, 3/6127) were found to be PCR positive at screening before hospitalization. The number of patients visiting the hospital and undergoing endoscopy were 2300/day and 1000/month, respectively. These patient volumes suggest that based on regional disease prevalence, one individual infected with SARS‐CoV‐2 may visit the hospital every day or undergo endoscopy every 2 months.

Using standard precautions helps prevent the spread of SARS‐CoV‐2 infections. In the endoscopy unit, we have been performing endoscopy according to the statement released from medical associations, including the Japan Gastroenterological Endoscopy Society. The major policies in that statement include rescheduling of endoscopic examinations if patients have an increased risk of having a SARS‐CoV‐2 infection, the use of standard precautions against COVID‐19, and “stay home” if HCWs have symptoms compatible with a SARS‐CoV‐2 infection. However, there were difficulties in fully complying with the statement because of an inadequate supply of personal protective equipment, insufficient PCR screening before endoscopy, and transfer of patients with emergency conditions and/or malignancies from other hospitals during the first wave of COVID‐19 (April–May 2020). The major route of spread of SARS‐CoV‐2 infections is by aerosol dispersion. We remind all HCWs to comply with standard precautions. As a result, the spread of SARS‐CoV‐2 infection was prevented in the endoscopy unit. In Northern Italy where the pandemic occurred, the number of HCWs infected with SARS‐CoV‐2 dramatically decreased after the adoption of routine standard precautions in the endoscopy unit.^3^ In addition, although one HCW contracted SARS‐CoV‐2 infection in the emergency department in our hospital, no other HCWs had SARS‐CoV‐2 infection using standard precautions during patient care encounters. These data support the effectiveness of standard precautions to prevent the spread of SARS‐CoV‐2 infection.

Although we found a low seroprevalence of SARS‐CoV‐2 antibodies among HCWs in the endoscopy unit, this study has acknowledged limitations. First, this is a single‐center study. Second, this study was conducted before the spread of variant strains of SARS‐CoV‐2, including the delta strain which is more easily transmitted than the original strain.^14^ Third, the accuracy of antibody measurement kits is not definitively known. The accuracy rate is extremely high according to information provided by the manufacturers, with 100% sensitivity and 99.8% specificity for the Roche assay and 100% sensitivity and 99.6% specificity for the Abbott assay. However, there were no patients with COVID‐19 around study subjects who tested positive for the antibody. In addition, no subjects tested positive as determined by all four antibody tests and the PCR test. This suggests the need for careful interpretation of the designation “antibody‐positive” in areas where COVID‐19 has a low prevalence.

In conclusion, the seroprevalence of SARS‐CoV‐2 antibodies among HCWs in the endoscopy unit was low through March 2021. The uses of triage, PCR screening, and adoption of standard precautions, were effective to prevent the spread of SARS‐CoV‐2 among HCWs in the endoscopy unit.

## CONFLICT OF INTEREST

Hironori Yamamoto received the provision of study materials from the SoftBank Group and Abbott Japan. Tatsuhiko Kodama received research funding from Murakami Foundation and Medical and Biological Laboratories Ltd, and provision of study materials from KOWA Company Ltd. Other authors declare no conflict of interest.

## FUNDING INFORMATION

Tatsuhiko Kodama received research funding from Murakami Foundation and Medical and Biological Laboratories Ltd.

## Supporting information


**Supplemental Table 1**. Questionnaire before endoscopic examinations

**Supplemental Table 2**. Personal protective equipment against SARS‐CoV‐2 in our hospital
