# Supplementary material for: Low prevalence of SARS‐CoV‐2 specific antibodies among endoscopists and their assistants in a university hospital in Tochigi prefecture–A single‐center study
Source: DEN Open. 2021 Dec 3;2(1):e79. doi: 10.1002/deo2.79 (PMC8828218; doi:10.1002/deo2.79)
Supplement: Supplementary file 1 — Supplemental Table 1. Questionnaire before endoscopic examinations [file DEO2-2-e79-s002.docx]

Supplemental Table 1 Questionnaire before endoscopic examinations

Temperature: ℃

1. Do you have any symptoms, including sore throat or cough?

2. Have you been in contact (suspected) with patients with Covid-19 within 2 weeks?

3. Have you been to foreign countries and/or endemic areas of Covid-19 within 2 weeks?

4. Do you have malaise or breathing difficulty?

5. Do you have an altered sense of taste?

6. Do you have diarrhea that has continued for 4 to 5 days?

The original version is written in Japanese.
