# Supplementary material for: Low prevalence of SARS‐CoV‐2 specific antibodies among endoscopists and their assistants in a university hospital in Tochigi prefecture–A single‐center study
Source: DEN Open. 2021 Dec 3;2(1):e79. doi: 10.1002/deo2.79 (PMC8828218; doi:10.1002/deo2.79)
Supplement: Supplementary file 2 — Supplemental Table 2. Personal protective equipment against SARS‐CoV‐2 in our hospital [file DEO2-2-e79-s001.docx]

Supplemental Table 2 Personal protective equipment against SARS-CoV-2 in the hospital

|  |  | Personal protective equipment | | | | |
| --- | --- | --- | --- | --- | --- | --- |
|  | HCW | Mask | Face shield | Glove | Gown | Cap |
| Endoscopy unit | PC | Surgical ^a)^ | Yes | Yes | Yes ^b)^ | Yes |
|  | Non-PC | Surgical ^a)^ | When needed | When needed | When needed | When needed |
|  |  |  |  |  |  |  |
| In/outpatient | PC | Surgical ^a)^ | When needed | When needed | When needed | When needed |
|  | Non-PC | Surgical ^a)^ | When needed | No | No | No |
|  |  |  |  |  |  |  |
| ICU | For COVID-19 | N-95 | Yes | Yes | Yes | Yes |

HCW, healthcare worker; PC, patient care; ICU, intensive care unit,

a) N-95 is required when seeing patients with COVID-19, b) Apron is added on during the shortage of gown.
